# Comparative Genome Analyses of 18 *Verticillium dahliae* Tomato Isolates Reveals Phylogenetic and Race Specific Signatures

**DOI:** 10.3389/fmicb.2020.573755

**Published:** 2020-11-30

**Authors:** Thomas W. Ingram, Yeonyee Oh, Tika B. Adhikari, Frank J. Louws, Ralph A. Dean

**Affiliations:** ^1^Department of Entomology and Plant Pathology, North Carolina State University, Raleigh, NC, United States; ^2^Department of Horticultural Science, North Carolina State University, Raleigh, NC, United States

**Keywords:** *Solanum lycopersicum*, secreted effectors, whole genome resequencing, soilborne fungus, *Verticillium dahliae* race 2

## Abstract

Host resistance is one of the few strategies available to combat the soil borne pathogenic fungus *Verticillium dahliae.* Understanding pathogen diversity in populations is key to successfully deploying host resistance. In this study the genomes of 18 *V. dahliae* isolates of races 1 (*n* = 2), 2 (*n* = 4), and 3 (*n* = 12) from Japan, California, and North Carolina were sequenced and mapped to the reference genome of JR2 (from tomato). The genomes were analyzed for phylogenetic and pathogen specific signatures to classify specific strains or genes for future research. Four highly clonal lineages/groups were discovered, including a lineage unique to North Carolina isolates, which had the rare MAT1-1 mating type. No evidence for recombination between isolates of different mating types was observed, even in isolates of different mating types discovered in the same field. By mapping these 18 isolates genomes to the JR2 reference genome, 193 unique candidate effectors were found using SignalP and EffectorP. Within these effectors, 144 highly conserved effectors, 42 mutable effectors (truncated or present in some isolates but absent in others), and 7 effectors present in highly variable regions of the chromosomes were discovered. Of the 144 core effectors, 21 were highly conserved in *V. alfalfae* and *V. longisporum*, 7 of which have no known function. Within the non-core effectors 30 contained large numbers of non-synonymous mutations, while 15 of them contained indels, frameshift mutations, or were present on highly variable regions of the chromosome. Two of these highly variable region effectors (HVREs) were only present in race 2 isolates, but not in race 3 isolates. The race 1 effector *Ave1* was also present in a highly variable region. These data may suggest that these highly variable regions are enriched in race determinant genes, consistent with the two-speed genome hypothesis.

## Introduction

*Verticillium dahliae* is a destructive soil-borne pathogen that infects hundreds of plant species (Bhat and Subbarao, 1999; Pegg and Brady, 2002; Klosterman et al., 2009). The pathogen can survive in soil for over a decade without a viable host present (Green, 1980; Xiao et al., 1998). Selected soil fumigants are widely used in vegetable fields as one of the few chemical-based strategies to suppress this pathogen (Gullino et al., 2002). Host resistance is a key management strategy for tomato growers (Klosterman et al., 2009) where race 1 predominates. Race 1 *V. dahliae* is characterized by the presence of a functional copy of the *Ave1* effector which is recognized by the host resistance gene *Ve1* (Diwan et al., 1999; de Jonge et al., 2012). Non-race 1 isolates (frequently described as “race 2”) have been discovered to be widely distributed in North Carolina since the 1980s (Bender and Shoemaker, 1984). Until recently the population of *V. dahliae* isolates infecting tomato were separated into race 1 and non-race 1 strains. Usami et al. (2017) revealed that new races could be distinguished amongst non-race 1 strains. Isolates that were pathogenic on tomato lines harboring the *Ve1* gene only, but found non-pathogenic on the tomato rootstock “Aibou” (aka “Aiboh” or V2) and “Ganbarune-Karis,” which contain an additional gene, were designated as race 2. Isolates pathogenic on “Aibou” and “Ganbarune-Karis” were described as race 3 (“non-race1, race2”). It was also shown that selfed “Aibou” resulted in progeny with a resistant to susceptible ratio of 3:1, indicating the race 2 resistance is conferred by a single dominant locus present in “Aibou” (Usami et al., 2017). In a more recent study, it was shown that “race 3” could be derived from the race 1 strain Vdp4 by knocking out the race 1 effector *Ave1* (Kano and Usami, 2019). These findings suggest that races 2 and 3 isolates may have emerged from different phylogenetic origins. Understanding diversity within pathogen populations is vital for successful breeding programs, as well as providing fundamental knowledge of strain evolution. There are currently no genomic sequences available of *V. dahliae* isolates from tomato that are also from the United States. The absence of genomic data from the United States presents a serious information gap for breeders and researchers.

The first layer of biochemical plant defense is achieved through the detection of pathogen associated molecular patterns (PAMPs) and microbe-associated molecular patterns (MAMPs) such as flagella and chitin (Jones and Dangl, 2006; Akamatsu et al., 2013; Newman et al., 2013: Hayafune et al., 2014). Upon detection of pathogen signal molecules, plants activate their immune systems through pattern triggered immunity pathways (PTI) via salicylic acid, reactive oxygen species, MAP kinase pathways, phytoalexin production, and other biochemical processes (Ebel, 1986; Howe et al., 1992; Raskin, 1992; Peumans and Van Damme, 1995; Kombrink and Somssich, 1997; Sharma et al., 2011). In *V. dahliae*, chitin-binding lysin motif (LysM) effectors sequester chitin and thus increase host susceptibility by preventing the activation of PTI (Kombrink et al., 2017). The LysM effector *VdPDA1* (polysaccharide deacetylase) recently characterized in *V. dahliae* is one of several effectors responsible for scavenging chitin pre-resistance gene recognition, which aids in hiding chitin from the plant’s PAMP recognition genes. A *PDA1* homolog was also discovered in *Fusarium* spp. and other *Verticillium* spp. (Gao et al., 2019).

Effector triggered immunity (ETI) is typically a much more aggressive defense response than PTI (Tsuda and Katagiri, 2010). Host resistance genes typically encode cytoplasmic proteins that contain nucleotide binding site leucine rich repeats (NBS-LRR), which can directly or indirectly recognize the presence of pathogen effectors (Belkhadir et al., 2004). In foliar tissue, ETI often results in the hypersensitive response (HR), which leads to localized cell death and eventually systemic acquired resistance (SAR) (Kombrink and Schmelzer, 2001). While ETI generally results in HR in plant foliage, very little is known about ETI in roots. The *Ve1* locus in tomatoes results in nearly complete immunity to strains containing the *Ave1* effector (de Jonge et al., 2012). Co-expression of the *Ave1* gene and *Ve1* gene in foliar tissue of *Nicotiana glutinosa* results in the hypersensitive response (HR) (Song et al., 2017). However, there is only fragmentary evidence that HR occurs in tomato roots (Sutherland, 1991). A comparison of *Ve1*+ and *Ve1*- plants infected with *V. dahliae* strains Le1087 (race 1) and Le1811 (non-race 1) indicates that many defense reactions such as phenylalanine ammonia-lyase (PAL) and other enzymes are differentially expressed in incompatible interactions in roots (Hu et al., 2019). It is unclear whether race 1 or 2 resistant plants are eliciting an HR response or a more complex resistance response more akin to PTI or the post-HR SAR (Hu et al., 2019).

Currently, there are two publicly available annotated *V. dahliae* reference genomes, JR2 (GCA_000400815.2) and VdLs17 (GCF_000150675.1). JR2 was isolated from a tomato plant in Canada, and sequenced using PacBio at 250x coverage, and the scaffolds have been assembled into 8 distinct chromosomes. The JR2 genome was annotated using a combination of *in silico* gene prediction and 35 fungal proteomes, which resulted in the prediction of 11,426 genes (de Jonge et al., 2012). VdLs17 was isolated from lettuce in California and was sequenced using Illumina next generation sequencing at 7× coverage and assembled to 55 scaffolds. The VdLs17 genome was annotated using a combination of manual curation, BLAST prediction, and *ab initio* gene prediction uncovering 10,535 genes (Klosterman et al., 2011). Genome annotation typically involves *ab initio* gene discovery through the *in silico* recognition of open reading frames (ORFs), and the input of messenger RNA data from RNA-sequencing projects (Campbell et al., 2014a,b). Both methods have their limitations as *ab initio* annotation has difficulties accounting for introns within coding sequences, and mRNA data relies on specific genes being expressed in large quantities at the time of sampling (Yandell and Ence, 2012). In contrast, whole genome re-sequencing relies on mapping reads from next generation sequencing (NGS) of different isolates to reference genomes and subsequent extraction of gene information, observing indels and nucleotide polymorphisms. However, mapping to a reference genome relies on the reference genome and the re-sequenced isolate genome being closely related. Gene prediction algorithms such as EffectorP and SignalP can be used to filter annotated genes. SignalP detects signal peptide motifs on the N-terminus of protein sequences that is required for secretion through the canonical secretion pathway to outside the cell. As the vast majority of effectors must be secreted from fungal cells to interact with their hosts, this is a powerful tool for recognizing candidate effectors (Melhem et al., 2013; Armenteros et al., 2019). However, many other non-effector proteins are secreted. To refine predictions of candidate effectors other prediction algorithms such as EffectorP have been developed. EffectorP was trained on 94 experimentally confirmed effector genes from a diverse set of fungal pathogens (Sperschneider et al., 2016). In 2011, 127 effectors were discovered (using SignalP 3.0 and EffectorP 1.0) on the *V. dahliae* VdLs17 genome and 112 on the *V. alfalfae* VaMs.102 genome (Klosterman et al., 2011). *V. dahliae* is an asexually reproducing ascomycete, with no known sexual stage (Usami et al., 2009; Short et al., 2014). Chromosomal rearrangement via transposable elements (TEs), random mutation, and horizontal gene transfer all contribute to *V. dahliae* diversity (Chen et al., 2018; Shi-Kunne et al., 2018). VdLs17 (non-race 1) and JR2 (race 1) have vastly different chromosomal arrangements despite having few nucleotide differences (de Jonge et al., 2013). More research is needed to elucidate diversity within *V. dahliae* populations to understand how evolutionary processes are affecting pathogenesis.

The main objectives of this study were to: (i) illuminate the phylogenetic structure, and characterize genomic features, such as mating type and recombination, of *V. dahliae* tomato isolates from Japan, California, and North Carolina using whole genome re-sequencing and (ii) identify core and divergent effectors, as well as those associated with specific races. By combining whole genome resequencing, and mining for candidate effector genes with macroscopic phenotypic data, we provide new insights into this pathogen’s diversity and pathogenicity factors.

## Materials and Methods

### Fungal Isolation

North Carolina isolates were obtained from *V. dahliae* infested tomato fields from Henderson, Jackson, Haywood, and Buncombe counties, all in the temperate, high elevation, growing region of western North Carolina. *V. dahliae* was isolated from infected tomato by surface sterilizing stem segments and placing them on Sorenson’s NP-10 media for 2 weeks at 26°C (Kabir et al., 2004). Spore suspensions were diluted to 1 × 10^2^ conidia per mL and streaked on potato dextrose agar (PDA, Difco Lab., Detroit, MI, United States). Single spore isolates were obtained by hyphal tip isolation from 3-day old single spore colonies. California isolates Le1087 (race 1) and Le1811 (race 2) were supplied by Dr. Krishna V. Subbarao at UC Davis. California isolates Ca70 and Ca36 were supplied by Suraj Gurung from Sakata Seed America, Inc., Salinas, CA. DNA from all Japanese isolates [GFCa2, To22, Vdp4, Vd141 (aka Ud-141), GFCB5, and HoMCF] was kindly provided by Dr. Toshiyuki Usami at Chiba University in Japan (Usami et al., 2017).

### DNA Extraction

Single spore isolates were grown on PDA for up to 10 days. Plugs from those plates were used to inoculate autoclaved 150 mL conical flasks filled with 50 mL of potato dextrose broth (PDB, Difco Lab., Detroit). After 4 days on PDB, the resulting mycelia was decanted into a 50 mL Falcon centrifuge tube and spun at 6000 rpm for 10 min. The supernatant was discarded, and the pelleted mycelia was dried under a laminar flow hood on autoclaved filter paper for 5–10 min. Pellets were frozen with liquid nitrogen and ground with a mortar and pestle to a fine powder. DNA was extracted using a phenol-chloroform extraction (Usami et al., 2007). High molecular weight and overall DNA quality was confirmed by North Carolina State University (NC State) Genome Sciences Laboratory (GSL) in Raleigh NC using an Agilent 2200 TapeStation and the Agilent 2100 Bioanalyzer (Santa Clara, CA, United States).

### Whole Genome Sequencing

Library preparation and sequencing took place at the NC State GSL. Genomic DNA libraries of Vdp4 and GFCa2 were prepared using Nextera DNA Flex Library Prep Kit from Illumina (San Diego, CA, United States). Vdp4 and GFCa2 genomes were sequenced at 813x and 167x coverage using MiSeq v.2 150 bp PE flow cell. Genome libraries from the 16 other isolates was prepared using TruSeq Nano LT DNA kit by Illumina (San Diego, CA, United States) and were sequenced at 20–30x coverage using MiSeq v3 300 bp PE flow cell.

### Mapping to Reference Genomes and Coding Sequence Extraction

Adapters were removed, paired-end reads were merged, and QC scores <10 were trimmed in Geneious (Biomatters Ltd., Auckland, New Zealand). The paired reads were mapped to the 8 chromosomes of the JR2 reference genome (BioProject Accession PRJNA175765) using the Geneious mapper (Biomatters, Ltd., Auckland, New Zealand) with the “only non-default” option turned on being “Find structural variants, short insertions, and deletions of any size”. Annotations were transferred to the consensus sequence at 75% similarity and a cost matrix of 65% similarity (5.0/−4.0). Coding sequences were extracted from the consensus sequence for each individual isolate sequence with a minimum coverage of 2×. Each coding sequence was translated in Geneious (Biomatters, Ltd., Auckland, New Zealand). This process was repeated for the same 18 isolates mapped to the 55 contigs of the VdLs17 reference genome BioProject PRJNA28529 NCBI.

### Secreted Effector Discovery

Coding sequences from the consensus sequence of each isolate were filtered through SignalP 5.0 to determine if a signal peptide was present on the N-terminus of the gene coding sequence (Armenteros et al., 2019). During SignalP filtering the signal peptide, a ∼25 amino acid long sequence, was removed. Secreted sequences were then filtered through EffectorP (Sperschneider et al., 2016). Candidate effectors with transmembrane helices were identified using TMHMM Server v. 2.0 and were removed (Moller et al., 2001).

### Phylogenetic Analysis

The consensus sequences of all 18 isolates and the reference genome JR2’s (PRJNA175765) chromosomes were individually aligned using Mauve (Darling et al., 2004). Gaps and ambiguous sequences were removed using Geneious (Biomatters, Ltd., Auckland, New Zealand). Segregating sites and recombining sites determined using DnaSP v5 (Rozas et al., 2003). MAT1-2 were identified if they mapped to the JR2 region where the gene is present. MAT1-1 were identified by mapping the sequences isolates to the AB505215 contig.

Eight chromosome consensus sequences were concatenated from the 18 isolates from this study, as well as 8 outgroup genomes from Gibriel et al. (2019) and the reference genome JR2 and were aligned using Mauve (Darling et al., 2004). Genetic distances were calculated using the Jukes-Cantor model (Jukes and Cantor, 1969). Resampling was completed using the bootstrap method with 1000 replicates. Pathogenicity on tomato confirmed in this study or in other studies using universally susceptible standard “Bonny Best” (Faino et al., 2015; Gibriel et al., 2019).

### Unassembled Reads Effector Discovery

The unassembled reads that did not map to the VdLs17 reference genome were assembled using the Geneious *de novo* assembler at the default settings in version 2020.1.0. Open reading frames (ORFs) were annotated to the assembled contigs. ORF settings were set a minimum nucleotide length of 100. All ORFs annotations were extracted and translated to amino acids in Geneious and those that did not start with an ATG were removed. Amino acid sequences were processed by SignalP v. 5.0, and EffectorP v. 2.0. Candidate effectors with transmembrane helices were identified using TMHMM Server v. 2.0 and were removed. Sequences were analyzed for secreted effectors specific to race 2 isolates.

### Pathogenicity Assay

Pathogenic race designations of 12 isolates were confirmed by inoculating 3-week-old differential tomato cultivars in the greenhouse. Three tomato cultivars tested were “Bonny Best” (universal susceptible), “Red Defender” (Ve1+/race 1 resistant, race 2 susceptible), and “Aibou” (Ve1+/race 1 resistant, race 2 resistant). Inoculum was prepared by growing each isolate on PDA for 1–2 weeks. Distilled de-ionized autoclaved water at ∼22°C was used to wash spores from PDA plates. Spores were filtered through a double layer of autoclaved cheesecloth and diluted to 1 × 10^7^ conidia mL^–1^. Ten mL of this spore suspension were injected into the soil ∼1 cm from the base of the plant stem using a 10 mL sterile pipette. Disease ratings from 0 to 5 (0 = 0; 1 = 1–20; 2 = 21–40; 3 = 41–60; 4 = 61–80; 5 = 81–100) for V-shaped foliar chlorosis/necrosis symptoms were recorded every 7 days for 45 days post inoculation. Wilting was rated on an identical scale. Each experiment contained 4 reps. Wilting and chlorosis/necrosis scores were used to calculate area under the disease progress curve (AUDPC) scores for each isolate/cultivar combination. Plants were planted in 530 mL plastic cups with 2-ply Sungro (70–80% Canadian sphagnum peat moss; 5–10% vermiculite plus dolomite limestone) potting soil. Plants were top watered for 2-weeks post inoculation. Two weeks post inoculation, holes were cut in the bottom of the cups, which were placed in trays 12 plants per tray with 2–5 cm of tap water. A 100 mL aliquot of fertilizer “Miracle-Gro Water-Soluble All-Purpose Plant Food” at a concentration of 1g/1000 mL was added to each tray once a week. Photosynthetic photon flux density was 251 μmol photon m^–2^ s^–1^ with a blue, green, and red percentages being 37, 37.1, and 25.9% respectively, and were on for 12 h each day. Temperatures ranged between 23 and 27°C, and relative humidity ranged between 30 and 55%. Additional pathogenicity assays were conducted with “Aibou” for six key isolates (Le1087, Ca36, NC86, JL5c, KJ14a, and VdLs17) and a water control. To determine if “Bowman” and or “Ganbarune-Karis” most likely contained the race 2 resistance gene they were inoculated in a complementary experiment with water, Ca36, NC86, and KJ14a using identical parameters.

### Gene Ontology (GO) Analysis

Full amino acid coding sequences of all potential effectors from the isolates mapped to the JR2 reference genome were analyzed using Blast2GO 5.2.5 (Conesa et al., 2005). Annotations were determined by blasting sequences against the BLASTP database with default settings. Sequences were scanned using EMBL-EBI InterPro. GO annotations were determined using default settings. GO descriptions were trimmed using GO slim generic. Effectors with GO annotations indicating the proteins were involved in eukaryotic transmembrane functions were removed.

## Results

### Race Designation of *V. dahliae* Isolates

Four experiments were conducted to determine race designations of isolates. In experiment 1, isolates tested were pathogenic on “Bonny Best,” and only Le1087 was non-pathogenic on “Red Defender” (race 1 resistant) (Supplementary Table 1). Le1087, Ca36, and TC18a were non-pathogenic on “Aibou” (race 1 and 2 resistant). NC86, FL10b, NC85, KJ14a, FF5a, FL7a, JL5c, FL9b, and VdLs17 were all pathogenic on “Aibou” (Supplementary Table 1). The water control showed no symptoms in all cultivars. In experiment 2, race typing on “Aibou” confirmed that Le1087 and Ca36 were non-pathogenic on “Aibou,” while NC86, JL5c, KJ14a, and VdLs17 were pathogenic on “Aibou” indicating they can be classified as race 3 (Supplementary Table 2). Experiment 3 demonstrated that Ca70, Le1811, and FL10b were also race 3 (Supplementary Table 3). Experiment 4 confirmed that the race 2 isolate Ca36 was non-pathogenic on Bowman and Ganbarune-Karis, and race 3 isolates were pathogenic on those cultivars (Supplementary Table 4). In sum, the data showed Le1087 is race 1, that Ca36, and TC18a are race 2, and NC86, FL10b, NC85, KJ14a, FF5a, FL7a, JL5c, Le1811, Ca70, and FL9b are race 3. VdLs17 is also pathogenic on “Aibou” (Supplementary Table 2). The race typing of Japanese isolates included in this study were Vdp4 (race 1, Vdp4Δ*Ave1* = race 3), GFCa2 (race 2), Vd141 (race 2), To22 (Race 2), GFCB5 (Race 3), HoMCF (Race 3) as documented in two separate publications (Usami et al., 2017; Kano and Usami, 2019).

### *V. dahliae* Genome Statistics

Genomes were sequenced using NGS by Illumina MiSeq. Vdp4 had a coverage of 813x (based on an estimated genome size of 32 Mbp) with 98.3% of the total 86,689,970 paired reads (150 bp paired end MiSeq) assembled to the JR2 reference genome. GFCa2 had a coverage of 167x, with 98.59% of reads mapping to the JR2 reference genome. The other 16 genomes had coverages ranging from 20× to 25× with 94.3–95.9% of the totals of 1,046,827 and 1,329,386 paired reads (300 bp paired end MiSeq) (Supplementary Table 5). It is noteworthy that the additional depth of sequence coverage of Vdp4 and GFCa2 added little improvement to the genome assemblies mapped to JR2. Coding sequences extracted from the 18 sequenced genomes based on presence in the JR2 reference genome ranged from 10896 to 11404 (Supplementary Table 6). Coding sequences with signal peptides ranged from 1025 to 1055, and effectors from 162 to 174 (Supplementary Table 6). Genome statistics based on comparison to the VdLs17 reference genome were similar and are shown in Supplementary Tables 7, 8.

### Genetic Diversity of *V. dahliae*

**FIGURE 1:**
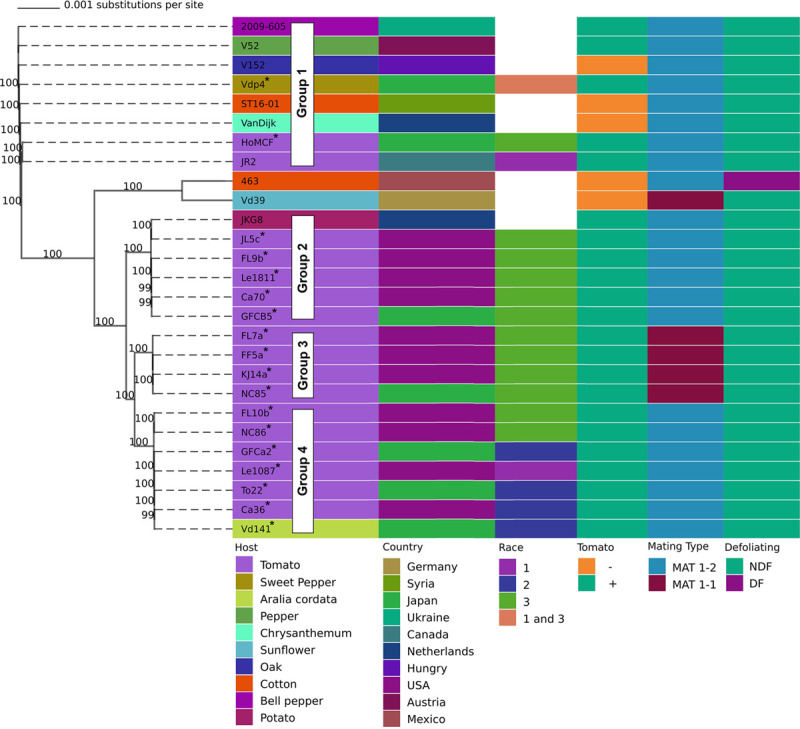
Phylogenetic tree developed from whole genome alignment of *V. dahliae* isolates from multiple hosts, countries, and mating types. Bootstrap values determined using 1000 replications. Isolates marked with * were sequenced for this study. Pathogenicity on tomato confirmed using universally susceptible tomato lines. Four phylogenetically distinct groups among isolates infecting tomato are indicated.

Based on whole genome phylogenetic analysis, isolates in this study were placed in to 4 distinct lineages/groups. Group 1 contained Vdp4 and HoMCF; Group 2: JL5c, FL9b, Le1811, CA70, and GFCB5; Group 3: FL7a, FF5a, KJ14a, and NC85; and Group 4: FL10b, NC86, GFCa2, Le1087, To22, Ca36, and Vd141 (Figure 1). VdLs17 is also in group 1 but was not included in Figure 1. Among the isolates in this study, strains classified as race 3 predominated Groups 2 and 3 and were also present in Groups 1 and 4. Within this study, Group 3 isolates were found exclusively in North Carolina, United States and were all MAT1-1. The only other MAT1-1 strain in our study was Vd39, an isolate from sunflower. Searching GenBank revealed the only other publicly available genome similar to Group 3 isolates is DVD-s29 from Canada, which is also a MAT1-1 strain.

### Genome and Effector Analyses

Across the 18 re-sequenced *V. dahliae* genomes, and the JR2 reference genome, 193 unique secreted effector regions were discovered using SignalP and EffectorP, and after sequences with transmembrane helices were removed (Table 1). Contained in all 19 genomes were 144 core effectors. There were 42 mutable effectors (MEs) that contained large truncations, large numbers of non-synonymous mutations, insertions, deletions, that caused them to fail being recognized as coding sequences in general, secreted proteins, or effectors. There were 7 effectors that were found in highly variable regions (HVREs), where locally collinear blocks (LCBs) were inconsistently maintained across all genomes (Table 1). Effectors were present on all chromosomes. Chromosome 1 contained the largest number of effectors at 37, while chromosome 3 had the fewest at 17. A total of 141,726 segregating sites containing nucleotide polymorphic sites were present on all genomes (Table 1). Across all chromosomes there were 481 sites of recombination, representing 0.33% of all segregating sites.

**TABLE 1:** Chromosome statistics of 18 isolates mapped to the JR2 reference genome. Segregating, recombining sites, and core/mutable/HVR effectors are displayed.

				**Effectors**			
**Chr.**	**Chr. Length**	**S^*v*^**	**RM^*w*^**	**Core^*x*^**	**ME^*y*^**	**HVRE^*z*^**	**Total**
1	9,275,483	15,604	109	32	5	0	37
2	4,277,765	17,442	57	14	6	3	23
3	4,168,633	20,833	51	9	8	0	17
4	4,086,908	20,751	90	20	5	2	27
5	4,171,808	16,321	28	15	3	2	20
6	3,530,890	21,065	62	18	5	0	23
7	3,277,570	12,895	44	17	3	0	20
8	3,361,230	16,815	40	19	7	0	26
Totals:	36,150,287	141,726	481	144	42	7	193

*^*v*^Polymorphic nucleotide sites on chromosome. ^*w*^Sites of nucleotide recombination on chromosome. ^*x*^Core effectors found in all 19 genomes with no insertions or deletions classes 1 and 2. ^*y*^Mutable effectors with indels or missing in some 1 or more isolate classes 3, 4, and 5. ^*z*^Highly variable region effectors (HVREs) classes 6, 7, and 8.*

### Gene Ontology (GO) Analysis of All Putative Effectors

**FIGURE 2:**
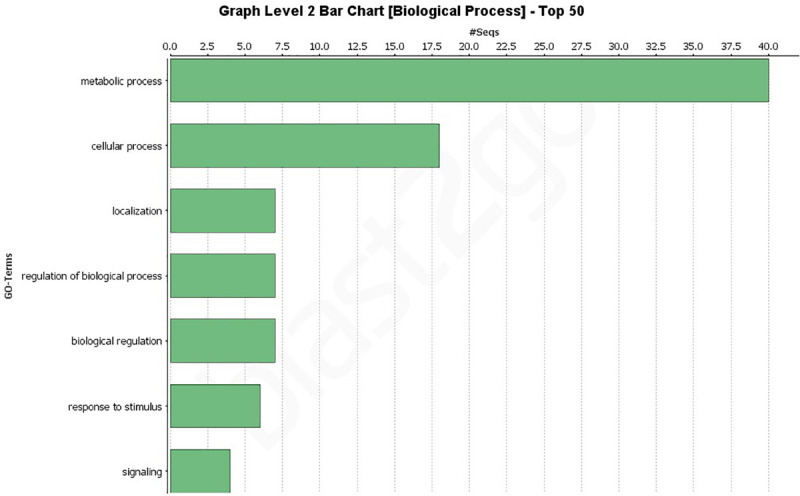
Gene Ontology (GO) level 2 bar chart of the biological processes of all 193 effectors found across 18 genomes and the JR2 reference genome. See Supplementary Figure 1 for full network analysis of all 193 effectors.

**FIGURE 3:**
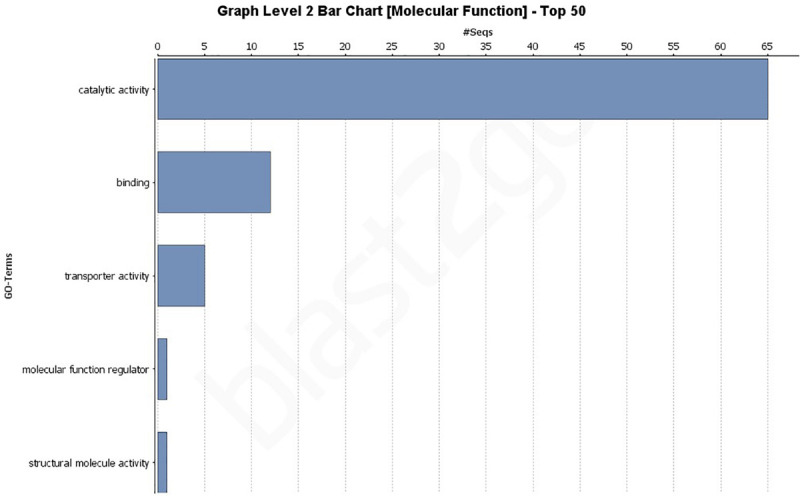
Gene Ontology (GO) level 2 bar chart of the molecular function of all 193 effectors found across 18 genomes and the JR2 reference genome. See Supplementary Figure 2 for full network analysis of all 193 effectors.

A GO analysis of all 193 effectors revealed a wide range of biological, molecular, and cellular component functions (Figures 2, 3 and Supplementary Figures 1–3). Simplified versions of the GO analysis are shown in Figures 2, 3, with complete versions in Supplementary Figures 1–3. The most common biological processes were metabolic processes and cellular processes representing 40 and 18 effectors, respectively (Figure 2 and Supplementary Figure 1) with at least 40 effectors involved in carbohydrate metabolic processes, and 13 involved in cellular macromolecule metabolic processes. In the molecular processes category, 65 effectors had catalytic activity, of those 33 had hydrolase activity, and 24 having lyase activity (Figure 3 and Supplementary Figure 2). There were 12 effectors that had binding activity, with 8 of those involved in nucleic acid binding (Figure 3 and Supplementary Figure 2). Sixty-three effectors had cellular component functions, with 45 possibly being cellular anatomical entities, and 34 of those having extracellular region functions (Supplementary Figure 3). These data revealed we have little current knowledge of the possible function of the majority of effectors while many of those with a possible function bind targets and have hydrolyzing ability.

### Classification and Genome Distribution of Effectors

The 193 effectors were broken down into 8 classes based on specific features (Table 2). Class 1 contained 75 core effectors (found in all 19 *V. dahliae* genomes) that had no non-synonymous mutations (NSM). There were 69 effectors classified as class 2 core effectors with 1 or more NSM. Class 3 contained 4 core effectors with truncation on the C-terminus in one or more isolates. There were 30 effectors classified as class 4 which contained non-core effectors that failed to pass through the SignalP or EffectorP pipelines in one or more isolates. Class 5 contained 8 effectors which had coding sequences missing in at least one genome. There were 4 effectors placed in Class 6 which were present in locally collinear blocks (LCBs) which were missing from one or more isolates. Class 7 contained a single effector (*Ave1*) present only in a highly variable region in an LCB only present in race 1 isolates JR2, Le1087, and Vdp4. The remaining 2 effectors were classified as Class 8 effectors and were present only race 2 isolates, and the race 1 isolates JR2, and Le1087 (Table 2). A full list of all unique effectors extracted from the 18 genomes and the JR2 reference genome, with their gene names and classification, is available in Supplementary Table 9.

**TABLE 2:** Unique effector-like regions discovered through filtering coding sequences through SignalP and EffectorP. Coding sequences were extracted from mapping reads from 18 *V. dahliae* genomes to the JR2 reference genome (ASM15067v2).

**Class**	**Effector groupings^*w*^**	**Number^*z*^**
1	Core effectors with no NSMs	75
2	Core effectors with 1 or more NSM	69
3	Core effectors with truncation	4
4	Other Non-Core effectors	30
5	Variable effectors	8
6	HVR effectors (HVRE) with no race specificity	4
7	HVR effector associated with race 1 only (Ave1)	1
8	Effectors only found in Race 2 Isolates, JR2, and Le1087	2
	Total	193

*^*w*^NSM = Non-Synonymous Mutations, HVR = Highly Variable Region. ^*z*^Individual effectors derived from mapping to the JR2 reference genome.*

Within the class 1 effectors, 21 effectors were highly conserved both in sequence and sequence lengths in all 19 genomes and *V. alfalfae* VaMs.102 (GCA_000150825) and *V. longisporum* (GCA_001268165). These cross-species core effectors display a diverse set of characteristics, 17 of which had GO descriptions (Table 3). In contrast, of the 15 genes assigned to classes 5–8 considered as non-core, only 8 had a specified GO description (Table 4). One of these non-core effectors has cysteine-rich domains found in CFEM-containing proteins (Kulkarni et al., 2003). Another is the race 1 effector *Ave1* (de Jonge et al., 2012).

**TABLE 3:** Core effectors (Classes 1 and 2) conserved in *V. dahliae, V. alfalfae* and *V. longisporum*.

**Gene ID**	**Description^*y*^**	**Length^*z*^**
Chr1g06580a	Predicted protein	234
Chr1g06610a-1	LAS seventeen-binding protein 1	195
Chr1g21900a-1	Metalloprotease	284
Chr1g27610a-1	Putative secreted protein	214
Chr2g00240a-1	Lysozyme	187
Chr2g00770a	Predicted protein	244
Chr4g09630a-1	Carbonic anhydrase	271
Chr5g00890a-1	Xylanase precursor	224
Chr5g09300a-1	Predicted protein	234
Chr5g09660a-1	Uncharacterized protein VDAG_05682	251
Chr6g03080a-1	Predicted protein	115
Chr6g06580a-1	Secretory phospholipase A2	187
Chr6g08770a-1	Necrosis- and ethylene-inducing protein and ethylene inducing peptide	233
Chr6g10190a-1	tRNA-specific adenosine deaminase 2	162
Chr7g00860a-1	Ccerato-platanin	138
Chr7g09430a-1	Carbonic anhydrase	271
Chr8g00170a-1	Dienelactone hydrolase family protein	261
Chr8g01530a-1	Pectin lyase	388
Chr8g05230a-1	Acyl-CoA dehydrogenase fadE12	164
Chr8g07470a-1	Predicted protein	164
Chr8g08300a-1	Signal recognition particle receptor subunit beta	198

*^*y*^Gene ontology description. ^*z*^Amino acid length.*

**TABLE 4:** Gene ontology and cellular localization analysis of effectors present in HVRs, or with missing coding sequences due to indels, of 18 *V. dahliae* genomes.

**Class**	**Gene ID**	**Description^*x*^**	**Length^*y*^**	**DLP^*z*^**
	Chr1g24730a-1	Putative hydrophobin precursor protein	258	Extracellular
	Chr1g27990a-1	Hypothetical protein BJF96_g6322	279	Extracellular
	Chr2g03590a-1	Predicted protein	66	Extracellular/nucleus
HVRE1	Chr2g09570a-1	—NA—	154	Extracellular
HVRE2	Chr2g10820a-1	40S ribosomal protein S26E	203	Extracellular
HVRE3	Chr2g10960a-1	Predicted protein	115	Extracellular
	Chr3g09700a-1	Putative secreted protein	191	Extracellular
	Chr3g10500a-1	—NA—	125	Extracellular
	Chr3g12130a-1	guanyl-specific ribonuclease f1	138	Extracellular
R2C2	Chr4g03650a-1	—NA—	60	Extracellular
R2C3	Chr4g03680a-1	Hypothetical protein VD0003_g10115	91	Extracellular
	Chr4g12040a-1	CFEM domain-containing protein	192	Cell membrane
Ave1	Chr5g02170a-2	EG45-like domain containing protein 2	134	Extracellular
HVRE4	Chr5g02460a-1	Folylpolyglutamate synthetase	252	Cytoplasm
	Chr8g02690a-1	Pantothenate transporter liz1	241	Extracellular

*^*x*^Gene ontology description. ^*y*^Amino acid length. ^*Z*^DLP = “DeepLoc localization prediction” of the target cellular region of the protein.*

### Predicted Effector Sequences Present in Race 2 Genomes

**FIGURE 4:**
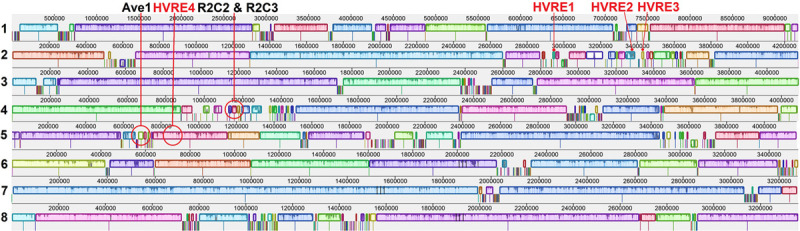
Mauve alignments of chromosomes 1–8 of JR2 with 18 genomes included in this study. Locally collinear blocks (LCBs) and highly variable regions (HVRs) are displayed. Shown here are the 3 race specific (black text) and 4 other effectors (red text) present in these HVRs on chromosomes 2, 4, and 5. Different colors are used to differentiate LCBs.

**FIGURE 5:**
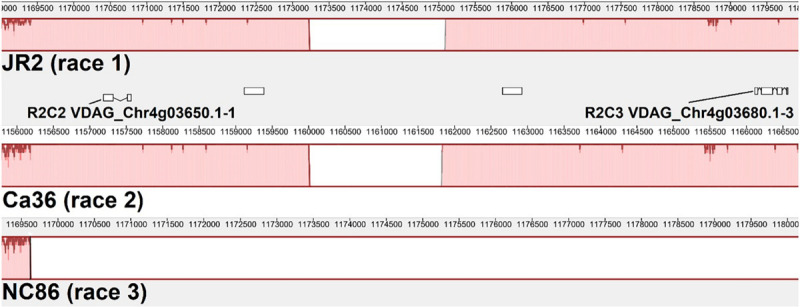
Mauve alignment of the specific region of JR2 chromosome 4 containing 2 race 2 candidate effectors (R2C2 and R2C3). Ca36 (race 2) and NC86 (race 3) are near-isogenic isolates belonging to group 4. Both candidate effectors are absent from NC86 and other race 3 isolates (not shown).

**FIGURE 6:**
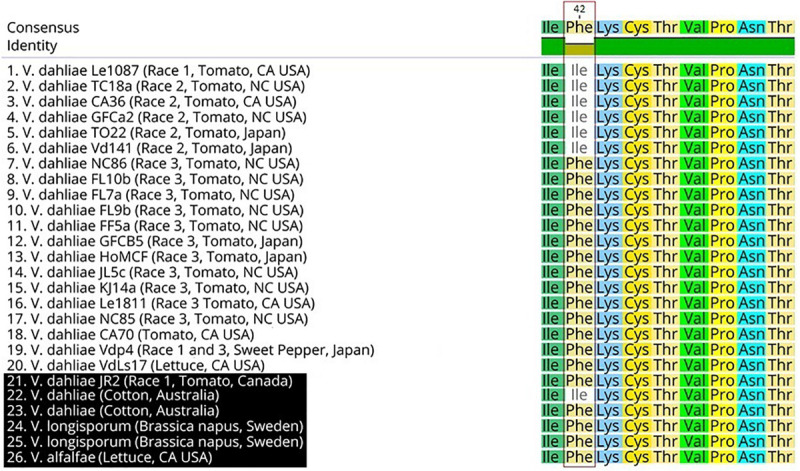
Protein sequence alignment of the R2C1 *VdPDA1* gene. Sequences highlighted in black (21–26) have not been tested for the race2/3 phenotype. A single nucleotide polymorphism of A to T results in an isoleucine (ATC) to phenylalanine (TTC) conversion.

Two race 2 candidate effectors were discovered using the JR2 reference genome (R2C2 and R2C3) (Table 5). In Mauve alignments different colors are used to differentiate locally collinear blocks (LCBs). LCBs are regions which are non-rearranged homologous regions present in more than one isolates genome. Both these effectors were found in highly variable regions of chromosome 4 (Figures 4, 5). The region containing these effectors was missing in the near isogenic group 4 race 3 isolates FL10b and NC86, but were present in race 2 isolates, JR2 (race 1, group 1), and Le1087 (race 1, group 4) (Figure 5). Within the analysis of effectors from the VdLs17 reference genome (a lettuce isolate pathogenic on race 2 resistant tomatoes), there was a single gene which contained a non-synonymous mutation found only in race 2 isolates, but not in race 3 isolates (Table 5 and Figure 6). This race 2 candidate (R2C1) is a LysM effector, *VdPDA1*, which binds to chitin to prevent the recognition of chitin by host defenses (Gao et al., 2019). Notably, *VdPDA1* was not recognized as a secreted effector in the JR2 reference genome because the provided annotation starts with a TTG start codon that adds 18 amino acids to the beginning of the gene sequence. JR2 contains the race 3 haplotype of *VdPDA1*, although its phenotype is unknown.

**TABLE 5:** Race 2 candidate effectors (R2C1-6) from three different discovery methods: VdLs17 reference genome; JR2 reference genome; and an *ab initio* gene discovery of the unassembled reads of the race 2 isolates mapped to the VdLs17 reference genome.

**Name**	**Gene ID**	**Method**	**Type^*w*^**	**JR2^*x*^**	**JR2 cDNA^*y*^**	**Le1087^*z*^**	**JR2 Chr4**
R2C1	*VdPDA1*	VdLs17 RG	NSM	–	–	+	1523795
R2C2	Chr4g03650.1-1	JR2 RG	Present	+	+	+	1170784
R2C3	Chr4g03680.1-3	JR2 RG	Present	+	+	+	1179336
R2C4	Contig 1 ORF 1	UR ORF	Present	+	–	+	1075295
R2C5	Contig 2 ORF 1	UR ORF	Present	+	–	+	1172205
R2C6	Contig 2 ORF 2	UR ORF	Present	+	–	+	1167261

*^*w*^Indicates how this gene is different between race 2 and race 3 isolates. NSM = gene present in both races but contains an NSM present only in race 2 but not in race 3. Present = gene and surrounding contig are only present in race 2 isolates, or race 1 isolates with no known race 2 phenotype (Le1087 or JR2) and are not present in race 3 isolates. ^*x*^Race 2 haplotype present in the JR2 genomic DNA. ^*y*^Race 2 haplotype present in the JR2 cDNA. ^*z*^Race 2 haplotype present in the Le1087 genomic DNA.*

### *De novo* Assembly of Unmapped Reads (Not Mapped to the VdLs17 Genome), Open Reading Frame (ORF) and Effector Analysis

Using an ORF analysis of a *de novo* assembly of reads that did not map to the race 3 reference genome VdLs17, three race 2 specific putative effectors (R2C4-6) were discovered (Table 5). The contigs containing these putative effectors were 29.4 Kb (contig 1) and 14.6 Kb (contig 2) in length. These putative effectors bore no sequence homology with known genes in BLAST and were not found in the JR2 cDNA library. Both contigs 1 and 2 resided in the highly variable region on JR2 chromosome 4 where R2C2 and R2C3 are located but were not annotated on that reference genome.

## Discussion

In this study, we present a first report of race 2 and 3 isolates in North America, as well as genome sequence and analyses of 18 *V. dahliae* of isolates from various races pathogenic on tomato from a wide range of geographic regions to reveal specific phylogenetic and race specific signatures. Isolates infecting tomato were grouped into four phylogenetically distinct lineages. Group 3 contained only mating type MAT1-1, while all other groups were MAT1-2. No significant evidence of recombination was observed on any of the chromosomes (Table 1). Of the 193 effectors identified using the JR2 reference genome, over 75% had few to no changes to their amino acid sequences among genomes, indicating their function may be necessary or they are involved in fitness of *V. dahliae* (Table 2). At least 21 effectors were highly conserved between *V. dahliae*, *V. longisporum*, and *V. alfalfae* (Table 3). Fifteen effectors had coding sequences missing from a number of isolates, with 7 of those effectors found in highly variable regions, and 2 of which were specific to race 2 (Tables 4, 5). We also found additional race 2 specific ORFs with effector signatures in assembled reads that did not align to the VdLs17 (race 3) reference genome.

### Phylogenetic Groupings

We found group 1 isolates to be the most phylogenetically distinct group, while the other groups were clustered much closer together (Figure 1). Group 1 also contains both reference genomes VdLs17 and JR2. According to other studies, VdLs17 and JR2 also have radically different chromosome structures, despite being phylogenetically similar to each other, having undergone chromosomal rearrangement in the not so distant past (de Jonge et al., 2013; Shi-Kunne et al., 2018). These data raise productive avenues of further research concerning the comparative chromosomal structure of groups 2, 3, and 4. Interestingly group 3 was only found in North Carolina, United States, and the only other publicly available genome comparable to group 3 was found from Canada (DVD-s29) which is also MAT1-1. An expanded analysis of a world-wide collection could ascertain the distribution of groups 1–4, potentially uncover additional groups, and provide a broader phylogenetic framework to offer additional insights regarding *V. dahliae* biology, race, and host range attributes. Further research is also needed into mating type genes, specifically group 3. While there is very little evidence of recombination, this may be due in part to the low number of MAT1-1, which make up only 1% of isolates, according to an analysis of 1120 isolates (Short et al., 2014). Group 3, which is unique to North Carolina and one location in Canada, has this rare MAT1-1 locus and is race 3. Interestingly several isolates of opposite mating types were found in the same fields, such as FL7a and FL9b, and NC85 and NC86 (Figure 1), although there is no evidence these isolates recombined in the recent past (Table 1).

### Secreted Effector Genes

In this study *V. dahliae* isolates pathogenic on tomato were shown to have between 162 and 174 secreted effectors using the JR2 reference genome (Supplementary Table 6). A study of the VdLs17 genome in 2011 showed only 127 effectors, however, the method by which they were classified as effectors was by sequence length (<400 bp) and being rich in cysteine (Klosterman et al., 2011). A more recent study of the VdLs17 (using SignalP v4.1 and EffectorP v1.0) reported 179 (Gibriel et al., 2019). In this study, additional secreted effectors were found. It is unclear if this increase in secreted effectors is due to the discovery of more true effector genes or more false positives. Phenotyping secreted effectors should be a priority to increase our understanding of these crucial genes. Both core and highly variable effectors may play vital functions that could be exploited by researchers to influence pathogenicity. At least 65 effectors were annotated as having some form of catalytic activity, 24 lyase, 33 hydrolase, and 15 oxidoreductase activity. This may indicate that a large number of these secreted effectors are involved in the metabolism or recognition of carbohydrates (Figure 3 and Supplementary Figure 2). Further analysis of these genes should be conducted to determine how many genes recognized as effectors are also carbohydrate active enzymes (CAZymes). In general, more in depth analysis of each individual secreted effector is warranted.

### Cross Species Core Effectors

Within the core effectors, 21 effectors were found not only in the 18 *V. dahliae* genomes in this study and the JR2 reference genome, but also *V. alfalfae* and *V. longisporum* (Table 3). Some of these highly conserved effectors have been shown to be important pathogenicity factors. Chr1g21900a-1 is a metalloprotease which is vital for *V. dahliae* virulence (Keykhasaber and Thomma, 2011). Chr6g08770a-1 is a necrosis- and ethylene-inducing protein and ethylene inducing peptide (VdNEP) which has been linked to wilting, and this gene is homologous to a similar gene in *Fusarium oxysporum* (Wang et al., 2004; Bae et al., 2006; Zhou et al., 2012). There are also six putative proteins with no known function (Table 3). More research is needed to identify the function of these predicted protein sequences. Some of these highly conserved core effectors may also be good targets for targeted host resistance research, such as host induced gene silencing.

### Highly Variable Region Effectors Found on the JR2 Reference Genome

While 15 effectors were completely absent from one or more isolates genome, seven of those were present in highly variable regions; one of which is the race 1 effector *Ave1*, and two of which were present only in race 2 strains. There were also three putative effectors found in the ORF analysis in these regions. Further research is needed into these race 2 candidate genes, specifically the 5 found in the highly variable regions. If any of the race 2 candidates found in highly variable regions are shown to be the true race 2 effector, this would add significant support the hypothesis that *V. dahliae* is operating under the two-speed genome hypothesis whereby “effector genes reside in genomic compartments that are considerably more plastic than the core genome” (Faino et al., 2016).

### *VdPDA1* and Other LysM Effectors

The only candidate effector with identified function is *VdPDA1*, which contains a chitin binding domain. Effector triggered immunity against LysM effectors is unknown in tomatoes. The non-LysM effector *Avr4* in *Cladosporium fulvum* protects chitin from hydrolysis by plant chitinases and can be recognized by host defenses in tomato (van den Burg et al., 2006). The *VdPDA1* gene may have chitin binding properties that are also found in other virulence factors of *V. dahliae* (Miya et al., 2007; Kombrink et al., 2017; Gao et al., 2019). A homologous *VdPDA1* (and functional) locus also exists in *Fusarium oxysporum* f. sp. *vasinfectum* which may function similarly, to the *V. dahliae* gene (Gao et al., 2019).

### Race Designations of *V. dahliae* Isolates

With the discovery of a new source of host resistance in tomato against *V. dahliae* race 2, new questions emerge about the nomenclature surrounding this pathosystem. Except for Vdp4 (races 1 and 3) and JR2 (race 1), all isolates in groups 1–3, as circumscribed in this study, are race 3 (i.e., can infect “Aibou”). *Ave1* is the effector responsible for the race 1 phenotype (de Jonge et al., 2012). Interestingly, in 2019 it was shown that deletion of *Ave1* from Vdp4 resulted in the ability to be pathogenic on “Aibou,” i.e., become race 3 (Kano and Usami, 2019). In contrast the race 1 isolate Le1087 is in the phylogenetically distinct group 4. Le1087 also contains all the race 2 candidate secreted effectors. Group 4 is the only group with race 2 isolates, which suggests that within this group race 2 evolved from race 1 by loss of the *Ave1* effector. Indeed, with the exception of Le1087, all isolates in group 4 lack *Ave1*. Further evidence for this hypothesis could be provided by deleting *Ave1* from Le1087 and evaluating pathogenicity on Aibou. Our results also call into the question the race nomenclature. Race 3 may simply represent isolates that lack races 1 and 2 effectors, which could mean that race 3 actually comprises many races. Race 3 and further race designations will require the identification of additional differential germplasm. Before the Usami et al., 2017 race 2 publication, many scientific publications referred to non-race 1 isolates as race 2.

## Conclusion and Perspectives

Whole genome analysis revealed 4 highly clonal lineages/groups of *V. dahliae*. Group 3 was unique to western North Carolina and has the rare MAT1-1 lineage. The recently described races 2 and 3 are both present in North Carolina and California in major tomato growing areas. Group 4 contains near-isogenic isolates of races 1, 2, and 3, indicating recent and rapid evolution. Many highly conserved cross-species effectors were observed. Using the JR2 reference genome there were seven effectors located in highly variable regions of the genome, the race 1 effector *Ave1*, and two race 2 specific effectors. In addition to those effectors, 4 other candidate race 2 effectors were identified using other methods.

To determine the nature of race 2 resistance in tomato, “Aibou,” and “Ganbarune-Karis,” were crossed and selfed, and their progeny were evaluated for resistance to race 2. The ratio of resistant to susceptible plants was 3:1 (Usami et al., 2017). This indicates that the resistance to race 2 is controlled by a single dominant locus. To maximize the genetic gain in this population, focus must be given to elucidate the plant-pathogen interactions at the molecular level. It appears that race 3 is widely prevalent in the region, thus, rapid and precision breeding is necessary by strategically integrating modern genomics approaches with advanced breeding techniques. Cost-effective third-generation sequencing technologies are now available to capture the SNPs via high-throughput genotyping. The identification and functional characterization of genes associated with disease resistance to all known races would be useful to ensure broad-spectrum and durable resistance to *V. dahliae.*

## Data Availability Statement

Raw data for this project is available through Sequence Read Archive (SRA) bioproject PRJNA640002, https://www.ncbi.nlm.nih.gov/bioproject/640002. Biosamples SAMN15297318–SAMN15297335.

## Author Contributions

TI conceived and designed the study with the guidance of YO, FL, and RD. TI performed the laboratory and greenhouse experiments. TI analyzed and interpreted the data with the aid of RD and YO. All authors contributed to the writing the manuscript. Grant writing and funding was provided by FL, TA, and RD. All authors have read and approved the final version of the manuscript.

## Conflict of Interest

The authors declare that the research was conducted in the absence of any commercial or financial relationships that could be construed as a potential conflict of interest.

## Supplementary Material

Supplementary Figure 1 Combined GO graph of the biological process of all 193 effectors.

Supplementary Figure 2 Combined GO graph of the molecular functions of all 193 effectors.

Supplementary Figure 3 Combined GO graph of the cellular component functions of all 193 effectors.

Supplementary Figure 4 Water control “Bowman” seedling 45 days post inoculation.

Supplementary Figure 5 Full amino acid alignment of *VdPDA1*.

Supplementary Figure 6 “Bowman” seedling 45 days post inoculation with race 2 isolate Ca36.

Supplementary Figure 7 “Bowman” seedling 45 days post inoculation with the race 3 isolate KJ14a.

Supplementary Figure 8 “Bowman” seedling 45 days post inoculation with the race 3 isolate NC86.

Supplementary Table 1 Experiment 1 of screening sequenced isolates against differential tomato lines. Bonny Best = universal susceptible; Red Defender = Ve1+ V2-; Aibou = Ve1+ V2+. Wilt and chlorosis/necrosis AUDPC scores displayed with Tukey’s HSD letters indicating significance groupings.

Supplementary Table 2 Experiment 2 of screening sequenced isolates against differential tomato lines. Bonny Best = universal susceptible; Red Defender = Ve1+ V2-; Aibou = Ve1+ V2+. Wilt and chlorosis/necrosis AUDPC scores displayed with Tukey’s HSD letters indicating significance groupings.

Supplementary Table 3 Experiment 3 of screening sequenced isolates against differential tomato lines. Bonny Best = universal susceptible; Red Defender = Ve1+ V2-; Aibou = Ve1+ V2+. Wilt and chlorosis/necrosis AUDPC scores displayed with Tukey’s HSD letters indicating significance groupings.

Supplementary Table 4 Experiment 4 of screening sequenced isolates against differential tomato lines. Bonny Best = universal susceptible; Red Defender = Ve1+ V2-; Bowman = Ve1+ V2+, Ganbarune-Karis (GBK) = Ve1+ V2+. Wilt and chlorosis/necrosis AUDPC scores displayed with Tukey’s HSD letters indicating significance groupings.

Supplementary Table 5 Total, assembled, and unassembled paired reads to the JR2 reference genome.

Supplementary Table 6 Total number of secreted coding sequence regions and secreted effectors present on the consensus sequence of reads mapped to the JR2 reference genome.

Supplementary Table 7 Total, assembled, and unassembled paired reads to the VdLs17 reference genome.

Supplementary Table 8 Total number of secreted coding sequence regions and secreted effectors present on the consensus sequence of reads mapped to the VdLs17 reference genome.

Supplementary Table 9 Spreadsheet of secreted effectors from JR2 RG analysis, and GO function definition.

Supplementary Table 10 Meta data of *V. dahliae* isolates sequenced in this study.
