# Supplementary figures and images for: Comparative Genome Analyses of 18 Verticillium dahliae Tomato Isolates Reveals Phylogenetic and Race Specific Signatures
Source: Front Microbiol. 2020 Nov 30;11:573755. doi: 10.3389/fmicb.2020.573755 (PMC7734093; doi:10.3389/fmicb.2020.573755)

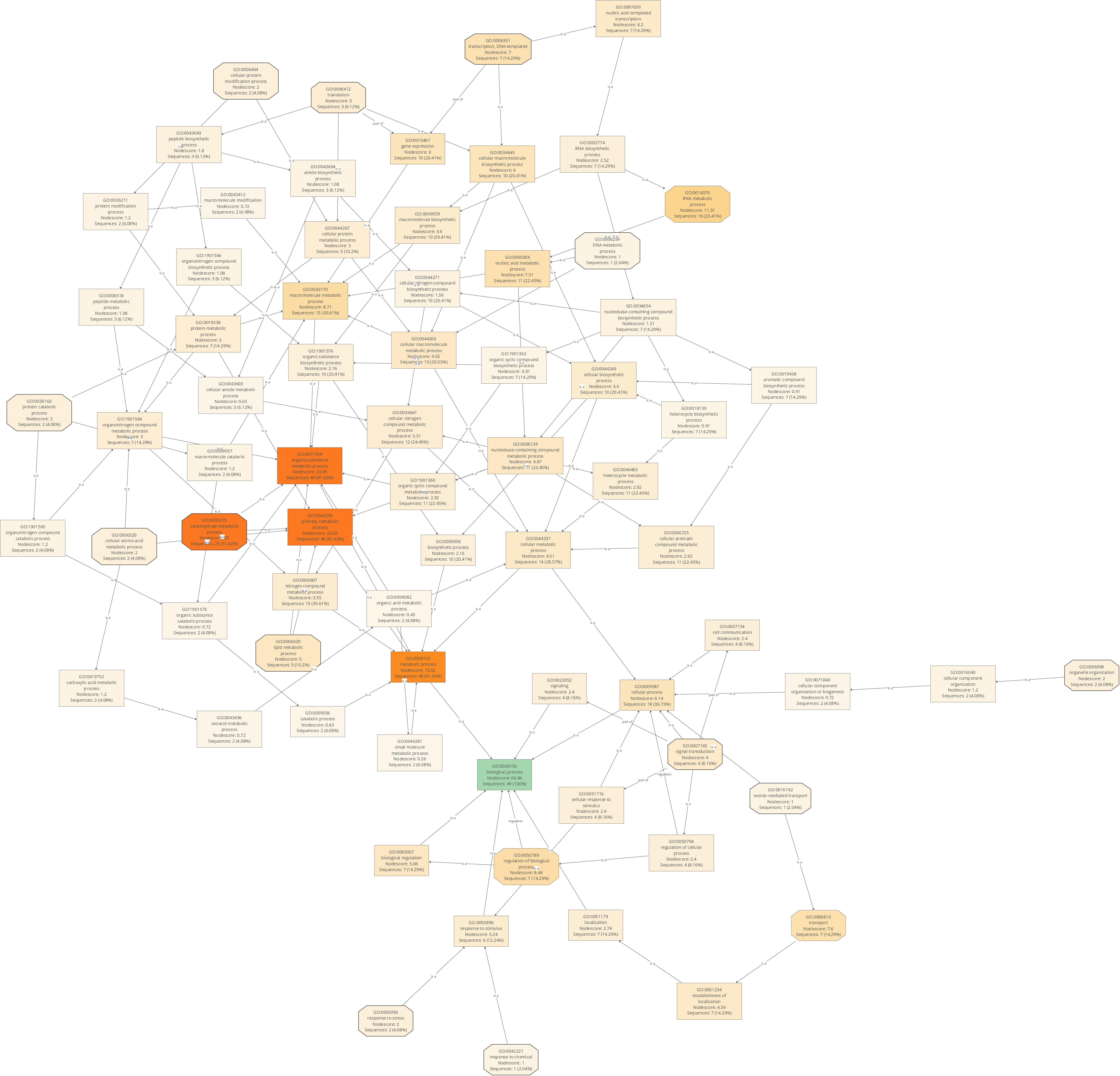

Supplement: Supplementary Figure 1 — Combined GO graph of the biological process of all 193 effectors. [file Image_1.PNG]

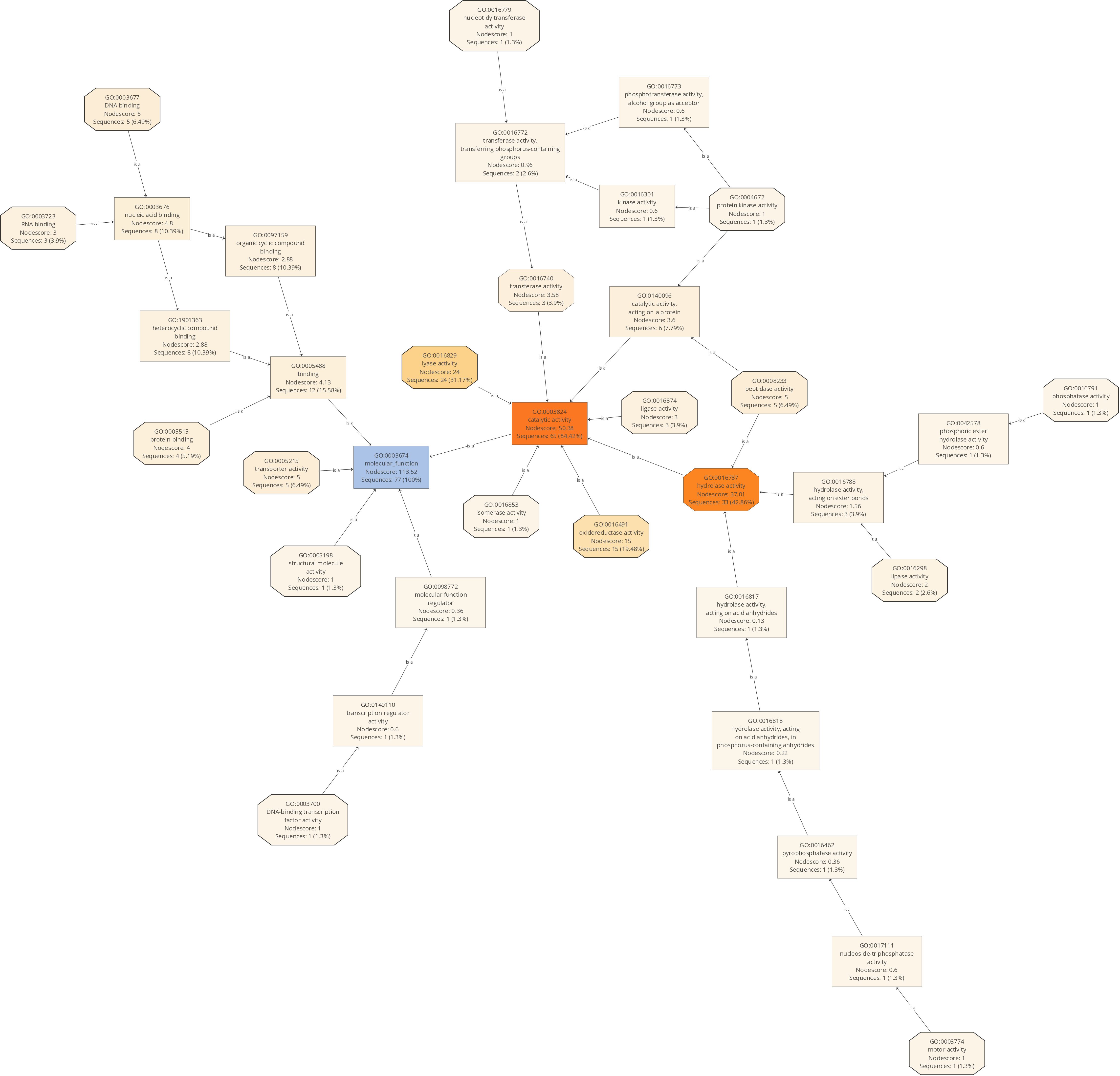

Supplement: Supplementary Figure 2 — Combined GO graph of the molecular functions of all 193 effectors. [file Image_2.PNG]

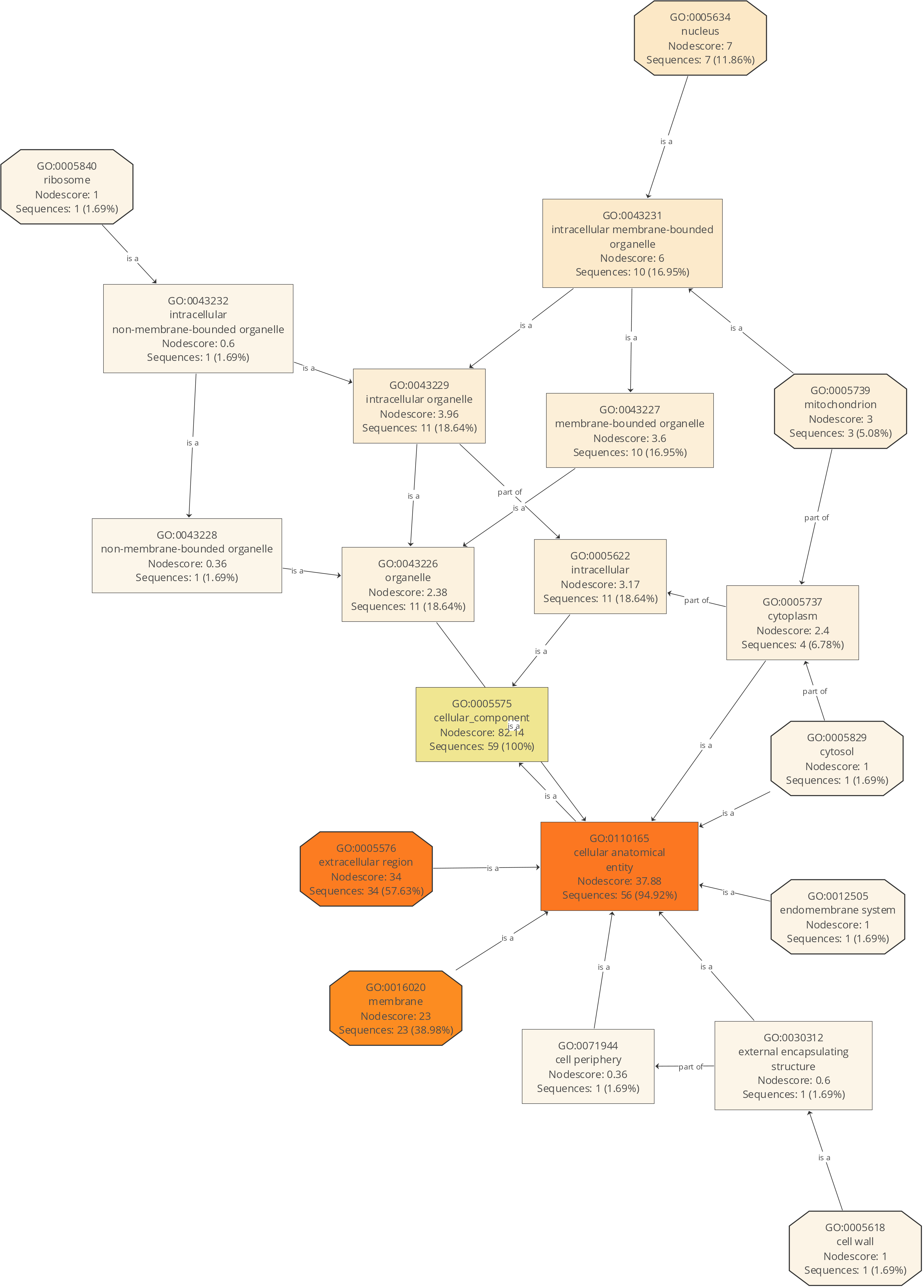

Supplement: Supplementary Figure 3 — Combined GO graph of the cellular component functions of all 193 effectors. [file Image_3.PNG]

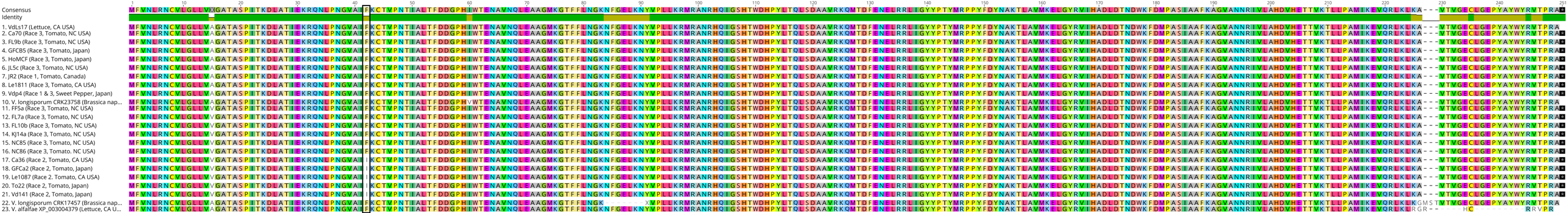

Supplement: Supplementary Figure 4 — Water control “Bowman” seedling 45 days post inoculation. [file Image_4.JPEG]

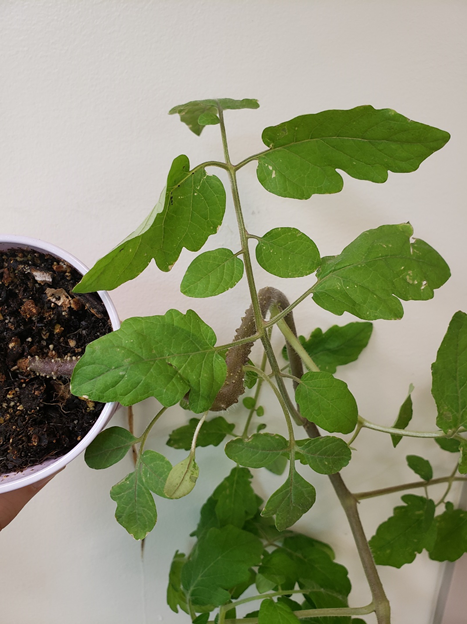

Supplement: Supplementary Figure 5 — Full amino acid alignment of VdPDA1. [file Image_5.PNG]

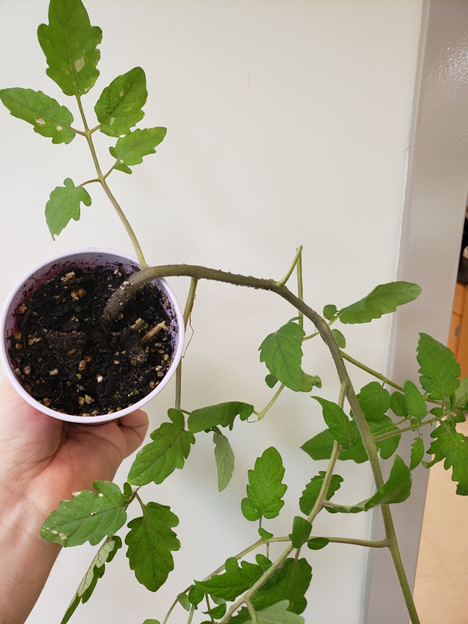

Supplement: Supplementary Figure 6 — “Bowman” seedling 45 days post inoculation with race 2 isolate Ca36. [file Image_6.PNG]

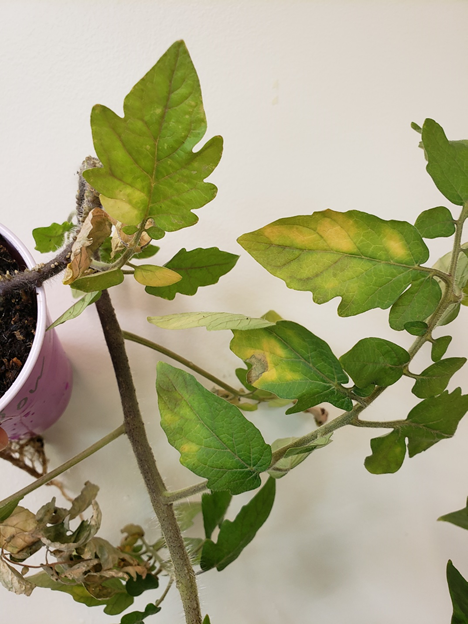

Supplement: Supplementary Figure 7 — “Bowman” seedling 45 days post inoculation with the race 3 isolate KJ14a. [file Image_7.PNG]

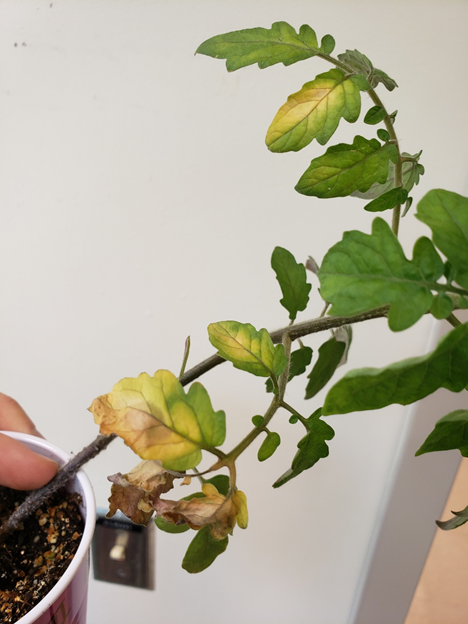

Supplement: Supplementary Figure 8 — “Bowman” seedling 45 days post inoculation with the race 3 isolate NC86. [file Image_8.PNG]
