# Supplementary material for: Comparative Genome Analyses of 18 Verticillium dahliae Tomato Isolates Reveals Phylogenetic and Race Specific Signatures
Source: Front Microbiol. 2020 Nov 30;11:573755. doi: 10.3389/fmicb.2020.573755 (PMC7734093; doi:10.3389/fmicb.2020.573755)
Supplement: Supplementary Table 1 — Experiment 1 of screening sequenced isolates against differential tomato lines. Bonny Best = universal susceptible; Red Defender = Ve1+ V2-; Aibou = Ve1+ V2+. Wilt and chlorosis/necrosis AUDPC scores displayed with Tukey’s HSD letters indicating significance groupings. [file Table_1.DOCX]

|  |  | Experiment 1 | | | | | | | | | | | |
| --- | --- | --- | --- | --- | --- | --- | --- | --- | --- | --- | --- | --- | --- |
|  |  | Bonny Best | | | | Red Defender | | | | Aibou | | | |
|  |  | Wilt | | CN | | Wilt | | CN | | Wilt | | CN | |
|  | Water | 0 | f | 0 | f | 0 | e | 0 | e | 0 | d | 0 | e |
| Group 1 | VdLs17 | 514 | c | 382 | d | 216 | c | 202 | cd | 96 | bc | 138 | bcde |
| Group 2 | FL9b | 386 | d | 308 | e | 174 | cd | 418 | ab | 234 | a | 226 | abc |
|  | JL5c | 340 | de | 340 | d | 124 | d | 138 | d | 92 | bc | 198 | abc |
| Group 3 | KJ14a | 432 | d | 418 | c | 404 | a | 418 | ab | 216 | ab | 322 | a |
|  | FF5a | 266 | e | 294 | e | 262 | bc | 414 | ab | 152 | bc | 212 | abc |
|  | FL7a | 450 | cd | 404 | cd | 358 | ab | 468 | a | 252 | a | 298 | ab |
|  | NC85 | 500 | c | 478 | c | 322 | b | 390 | ab | 60 | cd | 212 | abc |
| Group 4 | NC86 | 500 | c | 340 | de | 248 | c | 368 | ab | 64 | cd | 152 | cde |
|  | FL10b | 425 | d | 305 | e | 234 | c | 418 | ab | 32 | cd | 240 | abc |
|  | TC18a | 832 | b | 666 | b | 248 | c | 294 | c | 0 | d | 18 | de |
|  | CA36 | 408 | d | 432 | c | 376 | a | 482 | a | 0 | d | 0 | e |
|  | Le1087 | 1236 | a | 772 | a | 0 | e | 0 | e | 0 | d | 0 | e |

**Table S1**. Experiment 1 of screening sequenced isolates against differential tomato lines. Bonny Best = universal susceptible; Red Defender = Ve1+ V2-; Aibou = Ve1+ V2+. Wilt and chlorosis/necrosis (CN) AUDPC scores displayed with Tukey’s HSD letters indicating significance groupings.
