# Supplementary material for: Comparative Genome Analyses of 18 Verticillium dahliae Tomato Isolates Reveals Phylogenetic and Race Specific Signatures
Source: Front Microbiol. 2020 Nov 30;11:573755. doi: 10.3389/fmicb.2020.573755 (PMC7734093; doi:10.3389/fmicb.2020.573755)
Supplement: Supplementary Table 2 — Experiment 2 of screening sequenced isolates against differential tomato lines. Bonny Best = universal susceptible; Red Defender = Ve1+ V2-; Aibou = Ve1+ V2+. Wilt and chlorosis/necrosis AUDPC scores displayed with Tukey’s HSD letters indicating significance groupings. [file Table_2.DOCX]

|  |  | Experiment 2 | | | | | | | | | | | |
| --- | --- | --- | --- | --- | --- | --- | --- | --- | --- | --- | --- | --- | --- |
|  |  | Bonny Best | | | | Red Defender | | | | Aibou | | | |
|  |  | Wilt | | CN | | Wilt | | CN | | Wilt | | CN | |
|  | Water | 0 | d | 0 | e | 0 | d | 0 | d | 0 | c | 0 | b |
| Group 1 | VdLs17 | 322 | c | 284 | d | 244 | c | 180 | c | 294 | b | 238 | a |
| Group 2 | JL5c | 500 | a | 468 | b | 464 | a | 389 | b | 358 | a | 300 | a |
| Group 3 | KJ14a | 524 | a | 726 | a | 328 | b | 478 | a | 320 | ab | 280 | a |
| Group 4 | NC86 | 450 | ab | 418 | bc | 322 | b | 400 | ab | 280 | b | 240 | a |
|  | Ca36 | 425 | b | 340 | cd | 372 | ab | 150 | c | 0 | c | 0 | b |
|  | Le1087 | 538 | a | 460 | b | 0 | d | 0 | d | 0 | c | 8 | b |

**Table S2**. Experiment 2 of screening sequenced isolates against differential tomato lines. Bonny Best = universal susceptible; Red Defender = Ve1+ V2-; Aibou = Ve1+ V2+. Wilt and chlorosis/necrosis (CN) AUDPC scores displayed with Tukey’s HSD letters indicating significance groupings.
