# Supplementary material for: Comparative Genome Analyses of 18 Verticillium dahliae Tomato Isolates Reveals Phylogenetic and Race Specific Signatures
Source: Front Microbiol. 2020 Nov 30;11:573755. doi: 10.3389/fmicb.2020.573755 (PMC7734093; doi:10.3389/fmicb.2020.573755)
Supplement: Supplementary Table 3 — Experiment 3 of screening sequenced isolates against differential tomato lines. Bonny Best = universal susceptible; Red Defender = Ve1+ V2-; Aibou = Ve1+ V2+. Wilt and chlorosis/necrosis AUDPC scores displayed with Tukey’s HSD letters indicating significance groupings. [file Table_3.DOCX]

|  |  | Experiment 3 | | | | | | | | | | | |
| --- | --- | --- | --- | --- | --- | --- | --- | --- | --- | --- | --- | --- | --- |
|  |  | Bonny Best | | | | Red Defender | | | | Aibou | | | |
|  |  | Wilt | | CN | | Wilt | | CN | | Wilt | | CN | |
|  | Water | 0 | c | 0 | d | 0 | c | 0 | b | 0 | c | 0 | c |
| Group 2 | Le1811 | 322 | b | 364 | b | 442 | a | 478 | a | 400 | b | 474 | b |
|  | Ca70 | 382 | b | 300 | c | 290 | b | 354 | a | 340 | b | 446 | b |
| Group 3 | KJ14a | 364 | b | 478 | b | 538 | a | 492 | a | 538 | a | 616 | a |
| Group 4 | FL10b | 322 | b | 382 | c | 350 | ab | 474 | a | 400 | b | 542 | ab |
|  | Ca36 | 328 | b | 328 | c | 332 | ab | 464 | a | 0 | c | 0 | c |
|  | Le1087 | 940 | a | 816 | a | 0 | c | 0 | b | 0 | c | 0 | c |

**Table S3**. Experiment 3 of screening sequenced isolates against differential tomato lines. Bonny Best = universal susceptible; Red Defender = Ve1+ V2-; Aibou = Ve1+ V2+. Wilt and chlorosis/necrosis (CN) AUDPC scores displayed with Tukey’s HSD letters indicating significance groupings.
