# Supplementary material for: Comparative Genome Analyses of 18 Verticillium dahliae Tomato Isolates Reveals Phylogenetic and Race Specific Signatures
Source: Front Microbiol. 2020 Nov 30;11:573755. doi: 10.3389/fmicb.2020.573755 (PMC7734093; doi:10.3389/fmicb.2020.573755)
Supplement: Supplementary Table 4 — Experiment 4 of screening sequenced isolates against differential tomato lines. Bonny Best = universal susceptible; Red Defender = Ve1+ V2-; Bowman = Ve1+ V2+, Ganbarune-Karis (GBK) = Ve1+ V2+. Wilt and chlorosis/necrosis AUDPC scores displayed with Tukey’s HSD letters indicating significance groupings. [file Table_4.DOCX]

|  |  | Experiment 4 | | | | | | | | | | | | | | | |
| --- | --- | --- | --- | --- | --- | --- | --- | --- | --- | --- | --- | --- | --- | --- | --- | --- | --- |
|  |  | Bonny Best | | | | Red Defender | | | | Bowman | | | | GBK | | | |
|  |  | Wilt | | CN | | Wilt | | CN | | Wilt | CN | | | Wilt | | CN | |
|  | Water | 0 | d | 0 | c | 0 | b | 0 | c | 0 | b | 0 | b | 0 | b | 0 | b |
| Group 4 | Ca36 | 400 | b | 474 | a | 336 | a | 506 | a | 0 | b | 0 | b | 0 | b | 0 | b |
|  | NC86 | 272 | c | 336 | b | 276 | a | 400 | ab | 220 | a | 340 | a | ND | - | ND | - |
| Group 3 | KJ14a | 534 | a | 364 | ab | 368 | a | 350 | b | 294 | a | 294 | a | 166 | a | 372 | a |

**Table S4**. Experiment 4 of screening sequenced isolates against differential tomato lines. Bonny Best = universal susceptible; Red Defender = Ve1+ V2-; Bowman = Ve1+ V2+, Ganbarune-Karis (GBK) = Ve1+ V2+. Wilt and chlorosis AUDPC scores displayed with Tukey’s HSD letters indicating significance groupings. ND = strains were not determined on this cultivar
