# Supplementary material for: Comparative Genome Analyses of 18 Verticillium dahliae Tomato Isolates Reveals Phylogenetic and Race Specific Signatures
Source: Front Microbiol. 2020 Nov 30;11:573755. doi: 10.3389/fmicb.2020.573755 (PMC7734093; doi:10.3389/fmicb.2020.573755)
Supplement: Supplementary Table 5 — Total, assembled, and unassembled paired reads to the JR2 reference genome. [file Table_5.DOCX]

| **Table S5:** Total, assembled, and unassembled paired reads to the JR2 reference genome. | | | | | | | |
| --- | --- | --- | --- | --- | --- | --- | --- |
|  |  |  |  | Assembled Reads | | Unassembled Reads | |
| Isolate | Group^t^ | Paired Reads^u^ | Coverage^v^ | ANo.^w^ | A%^x^ | UNo.^y^ | U%^z^ |
| HoMCF | 1 | 1161501 | 22x | 1123919 | 96.76% | 37582 | 3.24% |
| Vdp4 | 1 | 8.7E+07 | 813x | 85204180 | 98.29% | 1485790 | 1.71% |
| Ca70 | 2 | 1046827 | 20x | 985540 | 94.15% | 61287 | 5.85% |
| FL9b | 2 | 1171506 | 22x | 1110799 | 94.82% | 60707 | 5.18% |
| GFCB5 | 2 | 1155626 | 22x | 1135977 | 98.30% | 19649 | 1.70% |
| Le1811 | 2 | 1203789 | 23x | 1152209 | 95.72% | 51580 | 4.28% |
| JL5c | 2 | 1198065 | 22x | 1129963 | 94.32% | 68102 | 5.68% |
| FL7a | 3 | 1132651 | 21x | 1078251 | 95.20% | 54400 | 4.80% |
| NC85 | 3 | 1272050 | 24x | 1205702 | 94.78% | 66348 | 5.22% |
| FF5a | 3 | 1238753 | 23x | 1157925 | 93.48% | 80828 | 6.52% |
| KJ14a | 3 | 1329386 | 25x | 1255407 | 94.44% | 73979 | 5.56% |
| NC86 | 4 | 1068019 | 20x | 996071 | 93.26% | 71948 | 6.74% |
| FL10b | 4 | 1137452 | 21x | 1056118 | 92.85% | 81334 | 7.15% |
| Ca36 | 4 | 1081891 | 20x | 984557 | 91.00% | 97334 | 9.00% |
| GFCa2 | 4 | 8627278 | 167x | 8505728 | 98.59% | 121550 | 1.41% |
| To22 | 4 | 1056938 | 20x | 1024208 | 96.90% | 32730 | 3.10% |
| Vd141 | 4 | 1128839 | 21x | 1099519 | 97.40% | 29320 | 2.60% |
| Le1087 | 4 | 1130435 | 21x | 1064004 | 94.12% | 66431 | 5.88% |
| ^t^Phylogenetic group | | | | | | | |
| ^u^Total paired reads from sequenced genome DNA of specified isolate | | | | | | | |
| ^v^Mean coverage of reads assembled to the JR2 reference genome | | | | | | | |
| ^w^Number of paired reads assembled to the JR2 reference genome | | | | | | | |
| ^x^Percent of paired reads assembled to the JR2 reference genome | | | | | | | |
| ^y^Assembled reads and percent of reads that were not assembled to the JR2 | | | | | | | |
| ^z^Percent of paired read that were not assembled to the JR2 reference genome | | | | | | | |
