# Supplementary material for: Comparative Genome Analyses of 18 Verticillium dahliae Tomato Isolates Reveals Phylogenetic and Race Specific Signatures
Source: Front Microbiol. 2020 Nov 30;11:573755. doi: 10.3389/fmicb.2020.573755 (PMC7734093; doi:10.3389/fmicb.2020.573755)
Supplement: Supplementary Table 6 — Total number of secreted coding sequence regions and secreted effectors present on the consensus sequence of reads mapped to the JR2 reference genome. [file Table_6.DOCX]

| **Table S6:** Total number of secreted coding sequence regions and secreted effectors present on the consensus sequence of reads mapped to the JR2 reference genome. | | | | |
| --- | --- | --- | --- | --- |
| Isolate | Group^w^ | Coding Sequences^x^ | Secreted^y^ | Effectors^z^ |
| JR2 | 1 | 11404 | 1055 | 174 |
| HoMCF | 1 | 10945 | 1028 | 169 |
| Vdp4 | 1 | 11233 | 1052 | 171 |
| Ca70 | 2 | 11035 | 1033 | 164 |
| FL9b | 2 | 11034 | 1036 | 165 |
| GFCB5 | 2 | 11025 | 1036 | 164 |
| Le1811 | 2 | 11033 | 1034 | 163 |
| JL5c | 2 | 10979 | 1030 | 165 |
| FL7a | 3 | 10948 | 1029 | 168 |
| NC85 | 3 | 10965 | 1032 | 162 |
| FF5a | 3 | 10960 | 1029 | 164 |
| KJ14a | 3 | 10952 | 1029 | 162 |
| NC86 | 4 | 10989 | 1026 | 167 |
| FL10b | 4 | 11008 | 1032 | 167 |
| Ca36 | 4 | 11005 | 1030 | 165 |
| GFCa2 | 4 | 11034 | 1036 | 165 |
| To22 | 4 | 11014 | 1033 | 165 |
| Vd141 | 4 | 10896 | 1025 | 164 |
| Le1087 | 4 | 11049 | 1030 | 165 |
| ^w^Phylogenetic grouping | | |  |  |
| ^x^Coding sequences extracted from consensus sequences of reads mapped to the JR2 reference genome | | | | |
| ^y^Total secreted genes extracted from consensus sequences of reads mapped to the JR2 reference genome | | | | |
| ^z^Total secreted effectors extracted from consensus sequences of reads mapped to the JR2 reference genome | | | | |
