# Supplementary material for: Comparative Genome Analyses of 18 Verticillium dahliae Tomato Isolates Reveals Phylogenetic and Race Specific Signatures
Source: Front Microbiol. 2020 Nov 30;11:573755. doi: 10.3389/fmicb.2020.573755 (PMC7734093; doi:10.3389/fmicb.2020.573755)
Supplement: Supplementary Table 7 — Total, assembled, and unassembled paired reads to the VdLs17 reference genome. [file Table_7.DOCX]

| **Table** **S7:** Total, assembled, and unassembled paired reads to the VdLs17 reference genome. | | | | | | | |
| --- | --- | --- | --- | --- | --- | --- | --- |
|  |  |  |  | Assembled Reads | | Unassembled Reads | |
| Isolate | Group^t^ | Paired Reads^u^ | Coverage^v^ | ANo.^w^ | A%^x^ | UNo.^y^ | U%^z^ |
| HoMCF | 1 | 1161501 | 22x | 1108494 | 95.4% | 53007 | 4.6% |
| Vdp4 | 1 | 86689970 | 813x | 83022801 | 95.8% | 3667169 | 4.2% |
| Ca70 | 2 | 1046827 | 20x | 996272 | 95.2% | 50555 | 4.8% |
| FL9b | 2 | 1171506 | 22x | 1104617 | 94.3% | 66889 | 5.7% |
| GFCB5 | 2 | 1155626 | 22x | 1098622 | 95.1% | 57004 | 4.9% |
| Le1811 | 2 | 1203789 | 23x | 1146068 | 95.2% | 57721 | 4.8% |
| JL5c | 2 | 1198065 | 22x | 1144093 | 95.5% | 53972 | 4.5% |
| FL7a | 3 | 1132651 | 21x | 1076825 | 95.1% | 55826 | 4.9% |
| NC85 | 3 | 1272050 | 24x | 1219284 | 95.9% | 52766 | 4.1% |
| FF5a | 3 | 1238753 | 23x | 1185698 | 95.7% | 53055 | 4.3% |
| KJ14a | 3 | 1329386 | 25x | 1262600 | 95.0% | 66786 | 5.0% |
| NC86 | 4 | 1068019 | 20x | 1012741 | 94.8% | 55278 | 5.2% |
| FL10b | 4 | 1137452 | 21x | 1077263 | 94.7% | 60189 | 5.3% |
| Ca36 | 4 | 1081891 | 20x | 1031070 | 95.3% | 50821 | 4.7% |
| GFCa2 | 4 | 26130444 | 245x | 24942431 | 95.5% | 1188013 | 4.5% |
| TO22 | 4 | 1056938 | 20x | 1004199 | 95.0% | 52739 | 5.0% |
| Vd141 | 4 | 1128839 | 21x | 1073598 | 95.1% | 55241 | 4.9% |
| Le1087 | 4 | 1130435 | 21x | 1075008 | 95.1% | 55427 | 4.9% |
| ^t^Phylogenetic group | | | | | | | |
| ^u^Total paired reads from sequenced genome DNA of specified isolate | | | | | | | |
| ^v^Mean coverage of reads assembled to the VdLs17 reference genome | | | | | | | |
| ^w^Number of paired reads assembled to the VdLs17 reference genome | | | | | | | |
| ^x^Percent of paired reads assembled to the VdLs17 reference genome | | | | | | | |
| ^y^Assembled reads and percent of reads that were not assembled to the VdLs17 | | | | | | | |
| ^z^Percent of paired read that were not assembled to the VdLs17 reference genome | | | | | | | |
