# Supplementary material for: Comparative Genome Analyses of 18 Verticillium dahliae Tomato Isolates Reveals Phylogenetic and Race Specific Signatures
Source: Front Microbiol. 2020 Nov 30;11:573755. doi: 10.3389/fmicb.2020.573755 (PMC7734093; doi:10.3389/fmicb.2020.573755)
Supplement: Supplementary Table 8 — Total number of secreted coding sequence regions and secreted effectors present on the consensus sequence of reads mapped to the VdLs17 reference genome. [file Table_8.DOCX]

| **Table** **S8:** Total number of secreted coding sequence regions and secreted effectors present on the consensus sequence of reads mapped to the VdLs17 reference genome. | | | | |
| --- | --- | --- | --- | --- |
| Isolate | Group^w^ | Coding Sequences^x^ | Secreted^y^ | Effectors^z^ |
| VdLs17 | 1 | 10535 | 1086 | 181 |
| HoMCF | 1 | 10138 | 1062 | 169 |
| Vdp4 | 1 | 10288 | 1068 | 170 |
| CA70 | 2 | 10149 | 1054 | 169 |
| FL9b | 2 | 10146 | 1059 | 170 |
| GFCB5 | 2 | 10148 | 1061 | 176 |
| Le1811 | 2 | 10153 | 1057 | 173 |
| JL5c | 2 | 10108 | 1056 | 174 |
| FL7a | 3 | 10082 | 1059 | 174 |
| NC85 | 3 | 10089 | 1058 | 172 |
| FF5a | 3 | 10094 | 1057 | 170 |
| KJ14a | 3 | 10088 | 1055 | 171 |
| NC86 | 4 | 10134 | 1054 | 171 |
| FL10b | 4 | 10142 | 1051 | 172 |
| CA36 | 4 | 10118 | 1053 | 172 |
| GFCa2 | 4 | 10151 | 1058 | 173 |
| TO22 | 4 | 10126 | 1052 | 173 |
| Vd141 | 4 | 10109 | 1048 | 175 |
| Le1087 | 4 | 10137 | 1054 | 171 |
| ^w^Phylogenetic grouping | | |  |  |
| ^x^Coding sequences extracted from consensus sequences of reads mapped to the VdLs17 reference genome | | | | |
| ^y^Total secreted genes extracted from consensus sequences of reads mapped to the VdLs17 reference genome | | | | |
| ^z^Total secreted effectors extracted from consensus sequences of reads mapped to the VdLs17 reference genome | | | | |
